# Genealogical analysis of European bison population revealed a growing up population despite very low genetic diversity

**DOI:** 10.1371/journal.pone.0277456

**Published:** 2022-11-11

**Authors:** Karolína Machová, Pavla Štruncová, Jan Calta, Ladislav Tichý, Luboš Vostrý

**Affiliations:** Department of Genetics and Breeding, Czech University of Life Sciences (CZU), Prague, Czech Republic; Natural History Museum of London, UNITED KINGDOM

## Abstract

In 1919, the European bison population became extinct in the wild. The rescue of the lowland subspecies and the whole species was achieved mainly thanks to individuals from the Białowieża Forest (Polish-Belarusian border). There are currently two breeding lines—the lowland (purebred *B*. *b*. *Bonasus*) founded by 7 individuals and the lowland-Caucasian (hybrids of *B*. *b*. *Bonasus* and *B*. *b*. *caucasicus*) founded by 12 individuals. This genealogical study was conducted on 15,071 individuals recorded in the pedigree book between 1881 and 2020. Its objective was to determine the level of genetic variability and inbreeding almost 100 years after the rescue measures were initiated. The completeness of the pedigree of the reference population was 77% in the fifth generation backwards. A maximum of 23 generations can be traced back in the pedigree. The average inbreeding coefficient and the mean average relatedness of the reference population were very high, about 17% and 16% respectively. No significant amount of new inbreeding was discovered. The reference population has lost 9.11% of the total genetic diversity compared to the population of founders. A male of the Caucasian subspecies Kaukasus was discovered among the ancestors of the lowland lineage reference population. The effective population size calculated based on the increase in inbreeding was 23.93 individuals, based on complete generations equivalent it was 16.1 individuals. Wright’s F-statistics showed very small differences in genotypic frequencies between individuals within the two lineages in the reference population (*F*_*IS*_ = 0.10), between individuals and the total population (*F*_*IT*_ = 0.04) and low differentiation between lineages (*F*_*ST*_ = 0.06). The population of the European bison from the Białowieża Forest is generally very uniform but still shows good fitness.

## Introduction

The Wisent or European bison (*Bison bonasus*) is Europe’s largest herbivore inhabiting almost the entire continent during the middle and late Holocene [1]. It belongs to the genus *Bovini* and is crossable with other genera Bos and Bison members, while fertility is preserved in female F1 hybrids [2]. Originally, there were two species of wild aurochs present in Europe since ancient times, in addition to the wisent, it was also *Bos primigenius*, which became extinct in 1627 [3]. Wisent had to face a similar fate. It became extinct in Western Europe in the eleventh century [1] and during the Middle Ages, its numbers continued to decline due to habitat fragmentation and hunting [4]. In the eighteenth century, its area of distribution was limited to only two areas—Białowieża Forest in present Poland and the Caucasian region in the Kuban region (Russia). Both populations were almost extinct after the First World War, and the last refuges remained zoos, the Ascania-Nova reserve in Ukraine, and populations of imported animals from Białowieża Forest in southwestern Upper Silesia [5]. In 1919, with the last individual killed in the Białowieża Forest, it became extinct in the wild.

In 1923, for the first time, a systematic effort appeared to preserve this species in two subspecies, Caucasian *B*. *b*. *caucasicus* and lowland *B*. *b*. *bonasus*. However, the catalogue of living animals already numbered only about 50 individuals, in which the Caucasian subspecies were represented by only one bull [6]. The next record speaks of a total of 69 animals, some of which were subspecies hybrids [7]. In 1932 the pedigree book was finally published [8]. By the end of the 1930s, it was already clear that not enough purebred animals were alive to keep the two purebred subspecies of wisent, so only the lowland subspecies and its hybrids with the Caucasian subspecies were preserved [9]. At the same time, efforts were made to create a supportive population formed by crossing surplus male wisents with American bison cows and gradually crossing back their offspring with wisents [10]. Unfortunately, some purebred herds were subsequently contaminated with American bison or even domestic cattle from hybrid animals. On the first of January 1947, there were only 97 purebred wisents in Europe [9]. However, all of these animals, which participated in reproduction after the First World War, originated from only 12 completely different pairs of allelic sets [11]. Several couples from the original seventeen founders only had a single offspring or a single inbred grandchild.

After the Second World War, a somewhat chaotic period of entries in the pedigree book began. Firstly, no records were kept at all during the war, secondly, part of the records was destroyed during the war by bombing (because of this, records continued to be kept from number 471) and thirdly, there were several years of delays between recording and issuing records [8]. During such a long time, some individuals managed to be born and die without being recorded. The first release of bison into the wild took place in 1952 in Białowieża Forest [12]. However, the number of animals has grown so much at that time, that the bison was reintroduced to several areas of eastern Europe in the 1960s - Russia, Ukraine, Lithuania, Byelorussia, Kirghizia [13–15]. The current wild populations are in Belarus, Bulgaria, Germany, Latvia, Lithuania, Poland, Romania, the Russian Federation, Slovakia, and Ukraine and include around 2,500 adults [16]. These wild populations are maintained naturally with minimal human intervention in reproduction (eg: in the form of the introduction of new animals). Their basic units are often meeting and mixing herds of females and young bulls, which dominant adult males join only for the purpose of reproduction [17]. In captive and semi-captive breeding facilities, bison reproduction is controlled by humans in the direction of sustainable variability, i.e., exchange of animals and mating of closely unrelated individuals.

With this specific development, the re-created wisent population was in the viewfinder of scientists from the beginning. Mainly due to the study of a high degree of inbreeding and its effect on fitness [11,18,19]. Above the primary studies based on the pedigree book, molecular genetic research based on the study of allozymes [20,21], nuclear genes [22–24], and the mitochondrial genome [25–27] began to predominate with the advent of the 90s of the 20th century. They all came to similar conclusions, namely that the genetic diversity of the bison is low, and this was also confirmed by more modern methods based on microsatellites [28–31] and SNPs [32–34]. In addition, some introgression of the Caucasian genes into one of the Białowieża Forest populations, which was considered a purebred lowland, was revealed [35].

The low variability of the bison genome can be attributed to the low number of founders—only 17 individuals. Of these, only one male Plebejer (No. 45) established the lowland lineage and five males the Caucasian-lowland lineage, of which only three (45 Plebejer, 100 Kaukasus, 15 Bergründer) also contributed with male offspring to the population [36]. The degree of inbreeding is 44% in the lowland lineage, while it is 26% in the lowland-Caucasian lineage [4]. Although the value of the average inbreeding coefficient is lower in the second lineage, there are apparent signs of inbred depression, namely shortening or lengthening of the neurocranium and narrowing of the splanchnocrania [18]. In contrast, no obvious signs of inbreeding depression have yet been recorded for the lowland lineage [36]. This may be due to the enhancement effect of genes that have resulted in a fitness increase of the species [37]. Or by the fact that the founders of the lowland lineage did not carry genes that would burden the population and thus reduce the fitness of the population [36].

However, we cannot underestimate possible inbreeding depression in the lowland lineage, even though it has not yet been recorded. The close relationship of individuals is a risk factor for example for genetically based diseases. One of them could be an inflammation of the penis and foreskin (posthitis), which was the cause of 8% male mortality in the western part of Białowieża Forest between 1952 and 2000 [4] and also appears in the lowland lineage [38]. The disease probably has a genetic predisposition based on the fixation of a harmful recessive allele [28]. However, no significant match was found between the MHC II genotype of DRB3 and the incidence of this disease [39]. Even the mitochondrial heteroplasmy found in animals in the Białowieża Forest does not appear to be related to the disease [40]. The first candidate genes were not identified until 2014 by Oleński et al. [41] on the 15th chromosome in the range of 2Mb. The same team of authors repeated the same genome-wide association study five years later on a larger number of animals, including clinical information on disease severity, evaluated by a more reliable statistical method and more strict quality controls for SNPs [38]. They discovered on chromosome 25 the candidate gene for posthitis coding protein periplakin (PPL) associated with skin development [38].

All this knowledge is difficult to apply to breeding without information from the pedigree book. However, extensive genealogical analysis of bison has not been performed for more than 20 years. This paper aims to describe the pedigree structure of a wisent based on pedigree books and to identify the main factors that affect the genetic diversity of the European bison and its possible losses.

## Materials and methods

The data were obtained through the Białowieża National Park, which has a pedigree book on its website registered since 1947 [42]. All information, date of birth, the inclusion of the new bison in the book, date of death, and all other changes (mother or father changes, sex specification) have been supplemented over the years (even respectively) to the books to the section of corrections and transfers. The pedigree dates to 1881 and contained 15,071 animals born until 2020. The reference population for genetic analysis was determined according to the list of all living bison, i.e., animals that could potentially give rise to future generations, as of 31 December 2020. The following populations were also used as reference populations for lineage comparison: REF 1—lowland-Caucasian lineage, REF 2—lowland lineage.

The completeness of the pedigree was determined by the maximum number of generations traced, defined as the number of generations between the individual and his farthest ancestor [43]. In addition, the complete generations equivalent (*CGE*) was calculated as the arithmetic mean of the sum of the expected genetic benefits of ancestors to the individual from the reference population [44], and the pedigree completeness index (*PCI*), which is a method based on the expected genetic contributions of maternal and paternal lineages to the reference population [45]. In this study, 5 generations back were considered for *PCI* computation.

The inbreeding coefficient of the individual was computed according to Meuwissen and Luo [46]. Average values for the birth year (only animals with a known date of birth were included) or generation were used to show the changes in inbreeding over time. The increase of inbreeding coefficient between the two generations in the whole population was calculated as a regression coefficient from the means of the individual inbreeding coefficients per maximum number of generations or *CGE* [47]. Furthermore, ancestral and new inbreeding were computed according to Kalinowski et al. [48] with the program Grain v2.2 [49,50]. As an additional indicator of inbreeding was chosen average relatedness, defined as twice the probability that two randomly selected alleles from a population are identical in origin [51]. It was obtained based on the kinship matrix of individuals from which are the *AR* (average relatedness) coefficients obtained as a line vector based on the equation described by Dunner et al. [52].

The probability of gene origin was computed on the reference population. The effective number of founders (*f*_*e*_) and ancestors (*f*_*a*_), the total number of founders and ancestors, as well as the founder genome equivalent (*f*_*ge*_) were computed. The *f*_*e*_ and *f*_*a*_ were calculated based on the method established by Lacy [53] and adjusted according to Boichard et al. [44]:

$$f_{e}=\frac{1}{\sum \left(p_{i}^{2}\right)},$$

$$f_{a}=1/\sum_{k=1}^{f}p_{k}^{2},$$

where p_i_ expresses the share of genes that the founder *i* contributed to the creation of the next generation of descendants; *p*_*k*_ is a marginal contribution of each ancestor *k* as its genetic contribution, which is determined by the proportion of genes it contributes, and this proportion (or part of it) has not yet been explained by the contribution of its ancestor selected before it. Founder genome equivalent (*f*_*ge*_) was estimated according to Caballero and Toro [54] as half the reciprocal of the average relatedness of the reference population.

In the next step, founders and ancestors with the biggest genetic contribution to the reference population were identified. Genetic variability losses were calculated as follows:

Loss of genetic diversity (1-*GD*) due to genetic drift and the bottleneck effect [55]:

$$GD=1-\frac{1}{2f_{ge}},$$

loss due to the unequal contribution of the founders [54]:

$${GD}^{*}=1-\frac{1}{2f_{e}}$$

and loss due to genetic drift (as the difference between the two previous ones).

Based on the birth dates of the animals, generation intervals were calculated, defined as the average age of the parents at the birth of their offspring used for reproduction [56]. The average age of the parents at the birth of the offspring was also computed. The parameter is calculated for four paths, namely: father-son, father-daughter, mother-son, and mother-daughter. Effective population size *N*_*e*_ was found in two ways. Firstly, by regressing the birth data for the whole population or a given reference population:

$$N_{e}=\frac{1}{2b},$$

where *b* is the individual inbreeding coefficient over the *CGE*. Secondly, through an increase in the inbreeding of an individual in a given population [57]:

$${\bar{N}}_{eF}=\frac{1}{2\bar{\Delta F}}$$

Wright’s F-statistic [58] was used to compare both wisent lineages, calculated according to Caballero and Toro [54,59] in program R using the package kinship2 [60] kinship matrix computation. The degree of individual inbreeding (*F*_*IS*_) relative to the subpopulation in which it is found was used to determine the predominance of heterozygosity or homozygosity due to non-random mating within the subpopulation. The fixation index (*F*_*ST*_) was used to determine the level of genetic variability between lineages. And the total inbreeding coefficient (*F*_*IT*_) was used to evaluate the loss/increase of genetic diversity in the population from an individual’s perspective.

All other genetic parameters and demographic data were processed in the program Endog 4.8 [47,61].

## Results

### Basic demographic data and completeness of the pedigree

The total number of individuals registered in the pedigree book from 1881 to 31 December 2020 was 15,071. Of these, 1,646 lacked all the necessary data (father, mother and sex). Beside of these, there were 116 individuals with no gender determination, mostly due to death at birth or shortly thereafter. Pedigree analysis was performed on 6,797 females and 6,512 males. From 1960 to 1980, the number of animals born doubled every ten years. Between 1960 and 1970, 1,244 calves were born, and between 1970 and 1980, the total number increased by 2,232 to 4,827. The reference population numbered 1,971 individuals, of which 1,195 were females, 773 were males and 3 individuals had unknown sex.

**Fig 1:**
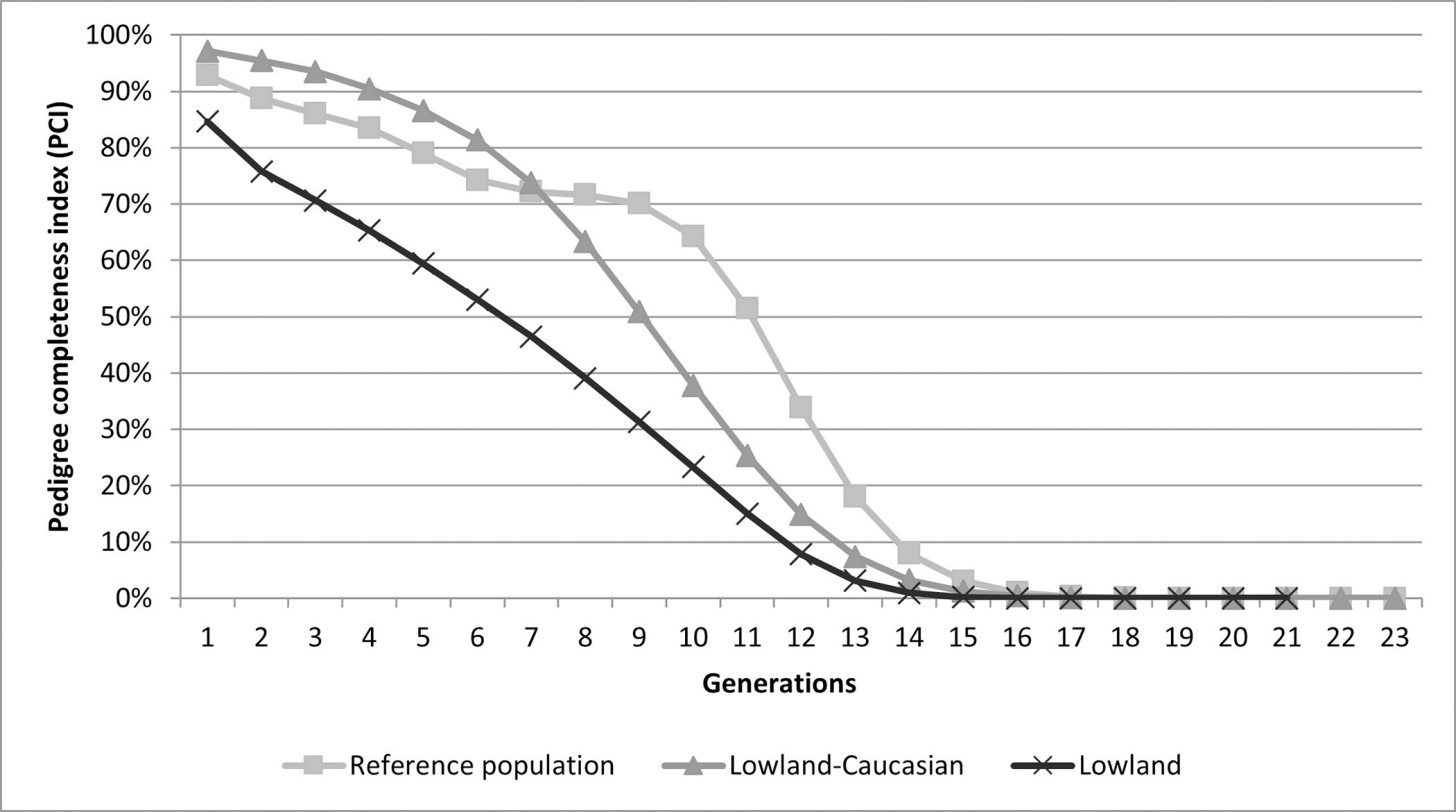
*PCI* values in maximum traceable generations for both lineages in the reference population.

A maximum of 23 ± 6.31 generations and 10 ± 2.55 complete generations could be traced in the reference population, which coincided with the results for the entire population. The mean *CGE* value for the reference population was 7.85 ± 3.65 (8.98 ± 3.61) before the inclusion records from 2020) and 6.72 ± 3.62 for the entire population. The *PCI* of the reference population for the last five generations was 90.08%, 88.74%, 86.20%, 83.64%, 77.26%. *PCI* was more than less constant from the 6^th^ to the 9^th^ generation back, but from the tenth generation it goes down sharply and before the 13^th^ generation, the pedigree contains only minimal information. Its values over the generations for the whole reference population and both lineages are shown in Fig 1.

From 1881 to 1920, 25 males out of 48 were used in breeding. As the population grew, so did the number of offspring per male and the number of males used in breeding. Since 1970, about 189 males have been used every ten years. Of the total number of males, 1,289 (19.80%) participated in reproduction, i.e., 5,222 males never reproduced until the end of 2020. The average number of offspring per reproducing male is 9.73 ± 10.53.

**Fig 2:**
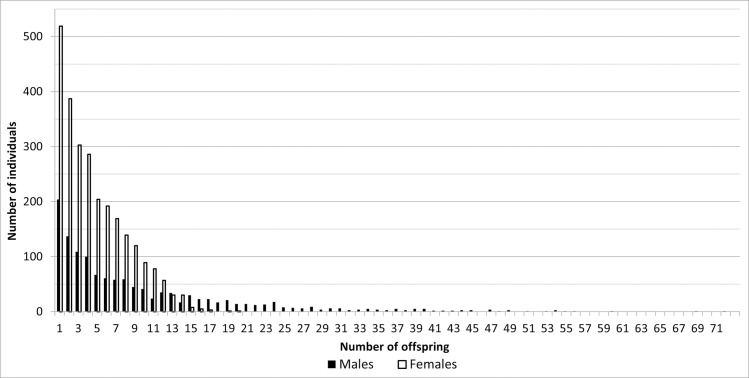
The number of males and females involved in reproduction with a given number of offspring.

A strong selection also occurred in females. Out of the total number of 6,795, 2,621 (38.57%) of them participated in reproduction, i.e. almost 4,174 did not reproduce until the end of 2020. The average number of offspring per female involved in reproduction is 4.74 ± 3.47. Most calves were born by a female 106 Friga (20 calves), who died in 26 years. Fig 2 shows the numbers of males and females involved in reproduction with different numbers of calves.

### Inbreeding coefficient and average relatedness

**Fig 3:**
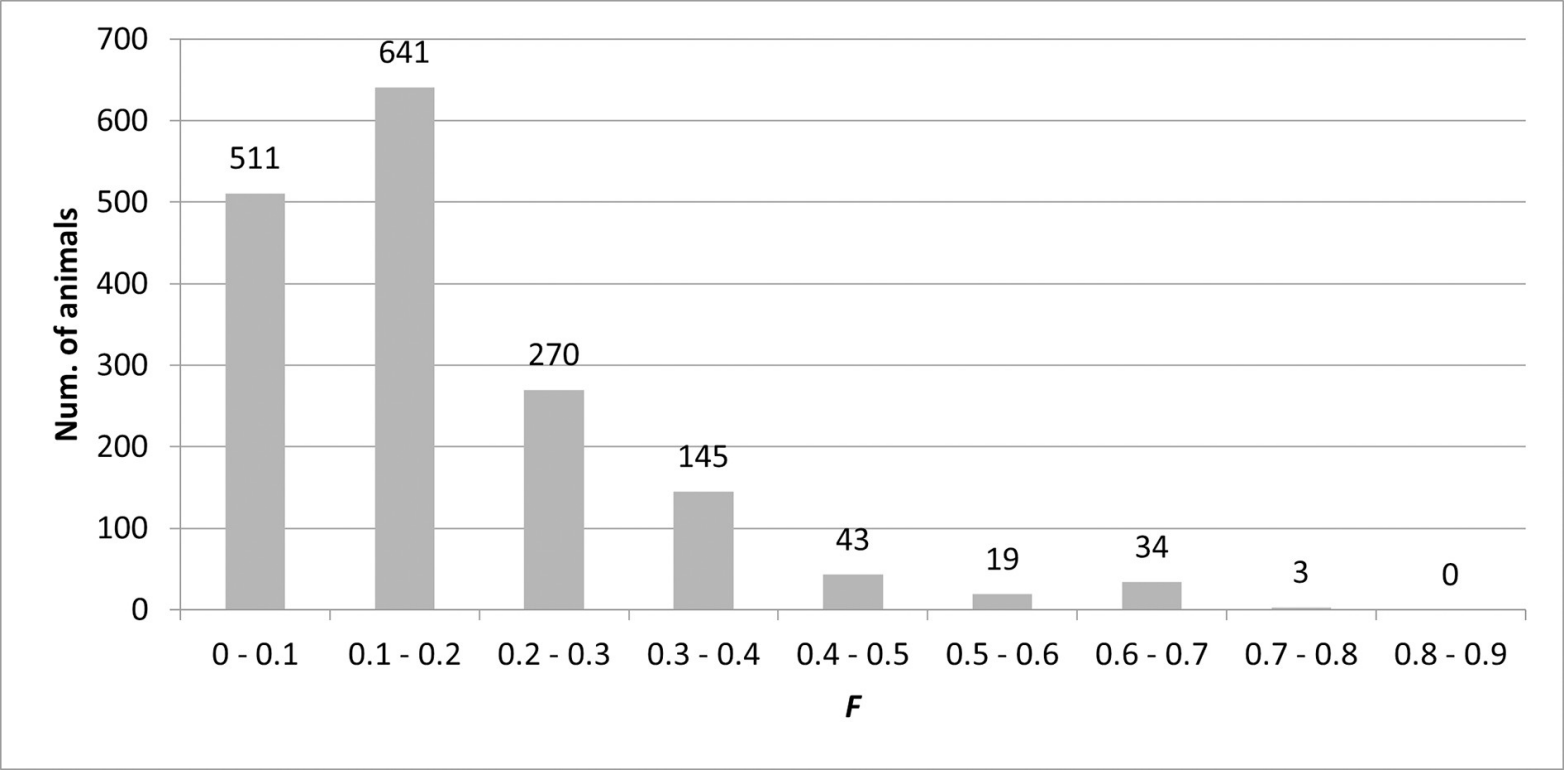
Range of inbreeding coefficient (*F*) in the reference population.

The highest value of the coefficient of inbreeding was 71.83% and was found in five animals, which were in the 15th generation. These were individuals with registration numbers: 12022, 12587, 12907, 13199, and 13872. Their *AR* to the whole pedigree was 27.90 ± 0.00%. Their parents are female Placka (11221) and male Plawiant (8940). The highest value of *AR* was 33.40% in the male Plisch (229). In the reference population, it was 28.14% in the male Sphinx (9054). The average value of *F* and *AR* for the whole population was 17.81 ± 15.36% and 16.07 ± 8.94% and for the reference population 17.02 ± 15.10% and 16.11 ± 7.96%. A comparison of *AR* and the average *F* for each generation is shown in Table 1. The distribution of the inbreeding coefficient in the reference population is shown in Fig 3.

**Table 1:** Average inbreeding coefficient and average relatedness in maximum generations.

Max. num. of gen.	Num. of animals	Mean *F* (%)	SD *F* (%)	The proportion of inbred animals (%)	Mean *F* of inbred animals (%)	SD F of inbred (%)	Mean *AR* (%)	SD *AR* (%)
**0**	2321	0.00	0.00	-	-	-	0.04	0.57
**1**	182	0.00	0.00	-	-	-	0.68	2.91
**2**	81	13.10	12.33	54.22	24.17	3.12	3.30	6.69
**3**	53	20.28	16.39	67.92	29.05	11.09	12.90	10.73
**4**	100	28.75	16.08	83.00	34.64	9.75	19.91	8.81
**5**	90	20.74	20.01	55.56	37.33	10.21	16.42	10.54
**6**	125	23.42	16.90	80.80	28.98	13.76	17.65	10.34
**7**	151	22.97	17.79	82.12	27.98	15.59	19.19	8.50
**8**	203	22.97	14.18	95.57	24.04	13.55	20.26	7.43
**9**	318	23.61	14.60	90.88	25.98	13.13	20.55	7.35
**10**	472	24.92	15.19	88.56	28.14	13.02	21.40	7.18
**11**	715	25.75	13.87	94.13	27.36	12.65	21.93	6.30
**12**	902	24.41	15.55	88.03	27.73	13.50	20.91	6.05
**13**	1109	24.30	15.22	91.34	26.60	13.86	20.43	6.03
**14**	1361	22.82	15.00	89.71	25.44	13.57	19.40	5.94
**15**	1326	22.04	15.60	87.48	25.20	14.09	18.92	5.53
**16**	1320	19.44	14.64	85.83	22.65	13.30	18.15	5.11
**17**	1124	20.73	12.61	95.02	21.18	11.98	18.95	4.35
**18**	1149	18.19	12.86	92.60	19.64	12.24	18.14	4.31
**19**	846	17.14	10.66	97.16	17.64	10.39	18.78	3.68
**20**	706	16.88	10.18	96.60	17.47	9.84	18.80	3.56
**21**	336	17.73	11.17	96.42	18.39	10.82	18.58	3.32
**22**	75	15.88	9.19	98.67	16.09	9.01	17.84	2.80
**23**	5	13.69	8.70	80.00	17.11	4.15	15.87	5.93

**Fig 4:**
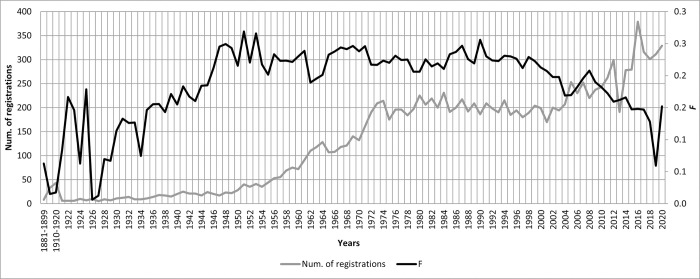
Coefficient of inbreeding (*F*) and number of individuals born between 1881 and 2020.

The most common range of the inbreeding coefficient was between 10–20% in 641 animals, and 511 animals had an inbreeding coefficient of up to 10%. No individual had this coefficient higher than 72%. A comparison of the development of the average inbreeding coefficient over time (1881–2020) with the number of registered animals is shown in Fig 4.

There was a 3% difference between ancestral inbreeding (*F*_*a-*Kal_) and total inbreeding (*F*). *F* had a value of 0.17 ± 0.15, while *F*_*a-*Kal_ had only 0.14 ± 0.13. There was no disproportionately large recent inbreeding present in the whole pedigree.

### Contribution of ancestors and founders to the reference population and loss of genetic diversity

The contribution of 17 founders to the reference population is shown in Table 2. The male 45 Plebejer (22.73%) and the female 42 Planta (15.05%) had the largest contributions. The total number of founders in the pedigree identified according to the definition of the founder [44], who participated in the reference population is 354. Male 4129 Kabir (2.90%), 3881 Kalvados (1.94%) and 4130 Kader (1.26%) and female 3882 Kamina (2.41%) also contributed to the reference population noticeably, but they were not among the first 17 original founders. Others accounted for less than 1%.

**Table 2:** Contribution of the original 17 founders to the reference population.

**Reg. num.**	**45**	**42**		**89**		**16**		**100**		**96**		**85**		**86**		**7**
**Contribution**	23.49%	15.56%		6.57%		4.65%		3.39%		3.20%		2.62%		2.62%		2.02%
**Reg. num.**	**95**		**1**		**2**		**32**		**33**		**46**		**123**		**122**	
**Contribution**	1.92%		1.01%		1.01%		0.73%		0.73%		0.64%		0.42%		0.25%	

For the reference population, the effective number of founders (*f*_*e*_) was 11 and the effective number of ancestors (*f*_*a*_) was also 11. The result of comparing these values (*f*_*a*_/*f*_*e*_) was 1, so no bottleneck effect was observed. The genome equivalent of the founders (*f*_*ge*_) of the entire population was 6.22 and 5.49 for the reference population. Only four ancestors would be needed to explain the 50% genetic contribution to the reference population. The total loss of genetic diversity of the reference population compared to the total population was found to be 9.11%. Of which, the loss caused by accidental genetic drift explains 4.56%, and 4.55% of the loss of genetic diversity is caused by the uneven contribution of the founders.

### Population recovery rate

The effective population size of the reference population calculated via the individual increase in inbreeding was 23.93 ± 4.26, and via regression on equivalent generations, it was 16.1. Generation intervals and the mean age of parents at the birth of the first offspring were in the reference population almost the same, 9.09 ± 3.94 and 9.08 ± 3.93 years, respectively. There were 722 animals in the reference population, whose offspring were already involved in breeding. The shortest generation interval in the reference population was from mother to son (8.56 ± 4.10 years), as well as in the whole population (8.69 ± 3.91 years). The mean age at birth of offspring was slightly lower in the whole population (8.98 ± 3.97) than in the reference population (9.08 ± 3.93). Both parameters (see Table 3) show high deviations from the mean (up to 4.25 years) in the reference population.

**Table 3:** Generation interval and mean age at birth of the offspring in the reference population.

	Num. animals	Interval (years)	Standard deviation (years)
**Generation interval**			
**Father-son**	164	8.86	± 3.76
**Father-daughter**	515	9.01	± 3.60
**Mother-son**	162	8.56	± 4.08
**Mother-daughter**	511	9.40	± 4.25
**Total**	1352	9.09	± 3.94
**Mean age at the birth of the first offspring**			
**Father-son**	579	8.84	± 3.67
**Father-daughter**	941	8.97	± 3.72
**Mother-son**	571	9.01	± 4.06
**Mother-daughter**	928	9.39	± 4.20
**Total**	3019	9.08	± 3.93

### Lineage comparison

The lowland lineage includes 3,735 individuals (1,737 males and 1,973 females), and the lowland-Caucasian 9,659 individuals (4,807 females and 4,750 males). Pedigree completeness showed slightly better results in the lowland-Caucasian lineage, 87% in the third generation and 77% in the fifth, while the lowland lineage showed 70% *PCI* in the third generation and only 59% *PCI* in the fifth (Graph 1). The average coefficient of inbreeding and the average relatedness do not differ much between the lineages. For the lowland lineage, *F* = 17.81% ± 15.36% and *AR* = 16.07% ± 8.94, for the lowland-Caucasian, *F* = 17.91% ± 15.35% and *AR* = 16.16% ± 8.89%. For animals from the reference population belonging to the lowland lineage, *F* = 17.02% ± 14.80% and *AR* = 16.42% ± 7.55%, for animals from the reference population belonging to the lowland-Caucasian lineage, *F* = 17.28% ± 15.16% and *AR* = 16.26% ± 7.89%.

The effective number of founders and ancestors of the lowland lineage was 7 and 7, respectively. The *f*_*ge*_ was 3.76. The genetic loss of this lineage was caused by 7.14% due to an uneven contribution of the founders and by 6.16% due to genetic drift. In total, it lost 13.30% of the total variability. For the lowland-Caucasian lineage, *f*_*e*_ and *f*_*a*_ were 11 and 9, respectively, and the effective number of the founders’ genomes was 4.32. The bottleneck effect is therefore noticeable in this lineage. The genetic loss for this lineage was calculated to be 11.58% and was caused also by both the uneven distribution of founders (4.55%) and genetic drift (7.03%). The largest contribution of the founders in both lineages was made by the male 45 Plebejer (lowland—33.53%, lowland-Caucasian—17.99%) and the female 42 Planta (lowland—18.62%, lowland-Caucasian—14.81%).

In the reference population of the lowland-Caucasian lineage, female 42 Planta (14.23%) and male 45 Plebejer (17.41%) were the most contributing founders. Other important founders were females 89 Bilma (9.77%), 16 Plavia (Musche) (6.09%) and 96 Gatczyna (5.06%). The contribution of the founding male 100 Kaukasus was 5.35%. The genetic variability of the reference population of this lineage could be explained by 164 ancestors. Of these, 229 Plisch (22.65%), 163 Borusse (12.73%), 87 Bill (Tor) (7.67%), 4457 Spratzer (6.17%) and 45 Plebejer (6.08%) were the most contributing male ancestors. Most contributing female ancestors were 89 Bilma (9.77%) and 106 Frigga (4.64%). The contribution of the original 17 founders to the lowland-Caucasian lineage is shown in Table 4A.

**Table 4:** a. The contribution of the original 17 founders to the lowland-Caucasian lineage part of the reference population. b. The contribution of the original 17 founders to the lowland lineage part of the reference population.

**Reg. num.**	**45**			**42**			**89**				**16**				**100**				**96**				**85**			**86**					**95**
**Contribution**	17.41%			14.23%			9.77%				6.09%				5.35%				5.06%				3.83%			3.83%					3.03%
**SD**	7.66%			5.71%			4.22%				2.45%				2.82%				2.10%				1.58%			1.58%					1.32%
**Reg. num.**	**7**				**1**				**2**				**32**				**33**				**46**				**123**				**122**		
**Contribution**	2.89%				1.44%				1.44%				1.16%				1.16%				1.01%				0.27%				0.16%		
**SD**	1.20%				0.60%				0.60%				0.82%				0.82%				1.00%				0.23%				0.14%		
**Reg. num.**		**45**			**42**			**16**				**89**				**86**				**85**				**123**			**7**			**122**	
**Contribution**		31.88%			16.50%			2.18%				1.12%				0.55%				0.55%				0.67%			0.55%			0.40%	
**SD**		20.40%			9.73%			2.05%				1.23%				0.60%				0.60%				0.64%			0.53%			0.38%	
**Reg. num.**			**1**			**2**				**33**				**32**				**96**				**95**				**100**		**46**			
**Contribution**			0.28%			0.28%				0.01%				0.01%				0.02%				0.01%				0.02%		<0.01%			
**SD**			0.26%			0.26%				0.08%				0.08%				0.23%				0.17%				0.32%		0.07%			

In the reference population of the lowland lineage, a very low contribution of male 100 Kaukasus was recorded, namely 0.02%. The genetic variability of the lowland lineage part of the reference population could be theoretically completely explained by 207 ancestors. Female 42 Planta (16.50%) and male 45 Plebejer (31.88%) were the most contributing founders, followed by the male Kabir contributed the most (7.14%). Other ancestors and founders accounted for less than 5%. The contribution of the original 17 founders to the lowland lineage is shown in Table 4B.

The total fixation index of *F*_*ST*_ is 0.06. which means that the monitored lineages are not genetically different. A positive *F*_*IS*_ value suggests that homozygous individuals may predominate in the population, but its value of 0.10 is also very low. The *F*_*IT*_ value was also low, only 0.04.

## Discussion

This study focuses on the whole registrated population of European bison (animals from enclosure-based centres, semi-wild breeding centres, or returned from freedom). Nearly 100 years have passed since the first rescue attempts. The size of the worldwide population has increased more than 23 times compared to the 84 purebred individuals living in early 1937 [9]. According to the IUCN Red List, in about a century, the European bison went from the category of extinct in the wild to the category of near threatened [16]. The maximum number of generations 23 is proportional to the age of the population since it is the longest line of ancestors that can be found in the family tree. The average age of the parents of the next generation would be somewhere between 4–5 years of age. However, the mean value of the equivalent of complete generations is significantly lower, almost 7.85 ± 3.65 *CGE* for the reference population and 6.72 ± 3.62 *CGE* for the whole pedigree. *CGE* simply tells how many ancestors are known. The smaller the number, the fewer ancestors are known. The *CGE* value we calculated is low even though it is certain, that all animals undoubtedly come from the same ancestors from Białowieża Forest [62], and thus it should be possible to trace all individuals back to these ancestors. The pedigree book has been struggling with errors in entries since the beginning of its existence. Although most of them are corrected with the release of the next part in the section "Additions and corrections to former lists", a certain degree of error can be assumed and naturally brings some limitations to our study. In addition, a lot of information is lost by removing some animals from the register due to a lack of information about the animals, or just by releasing them into the wild. Likewise, an animal from a freedom or semi-freedom breeding centre included in the register has no information about its ancestors and can therefore be considered a founder. Despite this, the quality of the European bison pedigree is comparable with pedigrees of production breeds of cattle. For example, for the reference populations of the Danish breeds Danish Holstein, Danish Jersey, and Danish Red, the following *CGE* values of 7.20, 7.36, 6.77 were observed and *PCI* values of 0.94, 0.9 and 0.93, respectively [63]. Not so high *CGE* was found in the breed Brown Swiss in Germany—6.24 [64], in Lidia cattle breed – 5.5 [65] or Normand cattle in Colombia– 5.21 [66].

The completeness of the pedigree also affects the coefficients used to analyze the origin of the genes, for example, the effective number of founders or the genome equivalent of the founders [44]. Their values are naturally very low in the European bison population due to its historical development. Its closed family tree has only 12 original genotypes since its inception. The variability of this population can therefore only decrease if we neglect the effect of a mutation. In this study, however, values related to founders are non-negligible influenced by individuals with unknown ancestors, who are understood as other founders by the program we used. In addition, errors in the pedigree, loss of alleles through genetic drift, and the assumption of absolute unrelatedness of ancestors affect the accuracy of the coefficients of gene origin. Therefore, the actual values will be even lower. Closed populations with such a small number of founding animals normally occur in the wild and some even go through in situ speciation [67]. However, the emergence of such populations is more often captured in new breeds of domesticated animals or laboratory strains, in the creation of which humans are directly involved [68–71]. The most common problem of such populations is severe inbred depression [72]. In some cases, however, the population is cleansed of strongly deleterious mutations due to a bottleneck, and the population is then still viable for hundreds of years [73]. This could hopefully be the case for the European bison, whose population shows no severe signs of inbred depression [36].

To identify losses of genetic variability caused by both the bottleneck effect and the uneven use of some individuals for breeding, the ratio between the effective number of ancestors and founders—*f*_*a*_ / *f*_*e*_—was used. Its 1 value shows no reduction in reproducing animals, and it could be logical because the first bottleneck effect cannot be seen in our data—the records of individuals living before that event do not exist. The second bottleneck effect caused by the Second World War is also not apparent. The presence of false founders is the main effect to blame, because, before the last pedigree book was included in the file, the value of this ratio was 1.13. The others are a low number of animals living at that time and an overall small number of founders. The *f*_*ge*_ / *f*_*e*_ ratio (0.50) suggests that random drift had a greater effect on the diversity of the reference population. The contribution of the founders to the reference population is very unbalanced. Most involved were Planta and Plebejer (15.05% and 22.73%) whose share since 1954 (18.8% Planta, Plebejer 26.4%) slightly fluctuates [11]. Founders 1, 2, 7, 32, 33, 46, 122, and 123 still contributed only minimally. Very surprising was the finding of a small relative share of the founder Kaukasus in the reference population of the lowland lineage. This is a significant finding that should be reviewed in the pedigree book for error. Otherwise, it would mean that the lowland lineage is not as pure as it was originally thought. However, the actual genetic contribution of the Kaukasus may have long since been swept away by genetic drift.

Regarding the values of the average inbreeding coefficient and average relatedness, the numbers we find differ from previous studies. The Olech and Perzanoski study [74] dealt with two herds in the Bieszczady, in the first herd in Stuposiana the *F* was 13.70%, in the second herd in Komańcza it was even 37.63%, and the average relatedness was 24.44% and 32.98%, respectively. Previous research focused on the diversity of both lineages also reached significantly higher values. Olech mentions values of nearly 50% in the lowland and 30% in the lowland-Caucasian individuals born 1996–2002 [75]. These findings indicate that the 17% inbreeding, which we measured in both lineages in the reference population, must be strongly underestimated in this context. A close relative of the European bison, the American Bison, has also undergone several bottleneck effects, and its current population was founded on less than 100 individuals [76]. However, the European bison shows lower genetic variability than the American bison, according to a study by Skotarczak et al. [77]. This was done on 4,269 American bison, with an inbreeding coefficient of 3.26% (17.81% for the European bison), with the highest value recorded at 46.87% (71.83% for the European bison). The value of the average relatedness was also very low—0.31%, compared to the *AR* of the European bison—16.07%. The absence of recent inbreeding indicates good breeding management. However, this fact must be taken with caution, due to the inclusion of individuals with unknown parents.

The effective size of the population is very small, the number of individuals that would cause the same increase in inbreeding as the reference population is only 23.93 animals. This coincides with the effective population size computed based on microsatellite analysis of 71 individuals born in the Białowieża Forest between 1996 and 2005, which was 28 [78]. This value is less than half the minimum effective size of 50 individuals, which is the goal of management to minimize inbreeding for the survival of the population in the short term [79]. A lower value was found for the American bison, namely, *N*_*e*_ = 11.64 [77]. Malhado et al. [80] also found a lower value for buffalo Jafarabadi, *N*_*e*_ = 10.4 ± 3.69. Despite the rapid growth of the bison population in recent decades, the value of *N*_*e*_ is very low and could hardly truly grow in the future. As with most other diversity parameters monitored here, its increase (or decrease in case of inbreeding) over the years is mainly due to the inclusion of animals from wildlife as founders, even though they share the same gene pool. The current bison population appears to be healthy. After nearly a century of inbreeding, this could be a purging effect of inbreeding or, in the case of herds kept in captivity for a long time, adaptation to captivity. This study included all types of breeding from free range to captive breeding, so determining the extent of these effects was not possible.

## Supporting information

S1 File (XLSX)
